# Peritraumatic startle response predicts the vulnerability to develop PTSD-like behaviors in rats: a model for peritraumatic dissociation

**DOI:** 10.3389/fnbeh.2014.00014

**Published:** 2014-01-28

**Authors:** Xinwen Dong, Yonghui Li

**Affiliations:** ^1^Key Laboratory of Mental Health, Institute of Psychology, Chinese Academy of SciencesBeijing, China

**Keywords:** post-traumatic stress disorder, startle, peritraumatic dissociation, vulnerability, rat models

## Abstract

Peritraumatic dissociation, a state characterized by alteration in perception and reduced awareness of surroundings, is considered to be a risk factor for the development of post-traumatic stress disorder (PTSD). However, the predictive ability of peritraumatic dissociation is questioned for the inconsistent results in different time points of assessment. The startle reflex is an objective behavioral measurement of defensive response to abrupt and intense sensory stimulus of surroundings, with potential to be used as an assessment on the dissociative status in both humans and rodents. The present study examined the predictive effect of acoustic startle response (ASR) in different time points around the traumatic event in an animal model of PTSD. The PTSD-like symptoms, including hyperarousal, avoidance, and contextual fear, were assessed 2–3 weeks post-trauma. The results showed that (1) the startle amplitude attenuated immediate after intense footshock in almost half of the stress animals, and (2) the attenuated startle responses at 1 h but not 24 h after stress predicted the development of severe PTSD-like symptoms. These data indicate that the startle alteration at the immediate period after trauma, including 1 h, is more important in PTSD prediction than 24 h after trauma. Our study also suggests that the startle attenuation immediate after intense stress may serve as an objective measurement of peritraumatic dissociation in rats.

## Introduction

Post-traumatic stress disorder (PTSD), triggered after exposure to a severe traumatic event, is a debilitating psychiatric disorder commonly characterized by the long-lasting hyperarousal, avoidance of reminders of the traumatic event, and involuntary re-experiencing of the trauma (American Psychiatry Association, 2000). Exposure to a traumatic event is necessary for the diagnosis of PTSD, but the post-traumatic symptoms become chronic only in a subgroup individuals exposed to trauma, at the rate around 25% (McFarlane, 2000; Ozer et al., 2003). Thus, it would be of great importance and challenge to identify the individuals who will subsequently develop full-blown PTSD after the traumatic event exposure. The acute responses in the immediate aftermath of traumatic events have been shown as a potential predictor of the development of PTSD (e.g., Yehuda et al., 1998; Bryant, 2006), along with the hypothesis that PTSD results from the abnormal traumatic memory formed during and immediate after traumatic exposure (Brewin et al., 1996; Brewin, 2001). Also, given some clinical evidence found that the early pharmacological or cognitive behavioral therapies have reduced the development of PTSD (Shalev et al., 2012; Steckler and Risbrough, 2012), the post-trauma prediction leads to a more effective prevention after trauma.

Peritraumatic dissociation, a typical acute stress response, has been reported as an effective predictor of the PTSD outcome among the early symptoms (e.g., Ursano et al., 1999; Van Loey et al., 2003). Some meta-analysis results found that peritraumatic dissociation had the largest effect size among pre- and post-trauma vulnerability factors of PTSD (Ozer et al., 2003; Breh and Seidler, 2007; Yufik and Simms, 2010), suggesting that it is one of the best predictors of PTSD.

However, the predictive power of peritraumatic dissociation has been challenged since the majority of peritraumatic dissociation studies were retrospective ones, in which the reports of peritraumatic dissociation were often inconsistent over time (David et al., 2010) and might be biased by the current severity of PTSD symptoms (Marshall and Schell, 2002; Candel and Merckelbach, 2004; Bryant, 2007). Thus, the prospective studies, assessing dissociation before PTSD is fully developed, are more convincing. Some prospective studies suggested that peritraumatic dissociation is an effective predictor of PTSD over time (e.g., Birmes et al., 2003; Schafer et al., 2006), whereas others did not (e.g., Marshall and Schell, 2002; Marx and Sloan, 2005). A meta-analysis even showed that only a few studies support that peritraumatic dissociation is an independent predictor of PTSD (van der Velden and Wittmann, 2008).

The inconsistence about the predictive ability of peritraumatic dissociation is most likely due to the various time points of its assessments. The dissociation symptoms subsided within a relatively short period and few would persist after 3 months post trauma (Van Loey et al., 2003), while these occurring around the time of trauma had little variance and predictive power (Cardena and Carlson, 2011). In addition, the effective time window of peritraumatic dissociation might be varied by the severity and type of trauma events, or demographic characteristics (van der Velden and Wittmann, 2008). In clinical reports, the effective time points for peritraumatic dissociation included 24 h (Michaels et al., 1999; Bennett et al., 2002; Birmes et al., 2003) and 1 week (Shalev et al., 1996; Van Loey et al., 2003), while the dissociation at 1 month after trauma did not significantly correlated with subsequent development of PTSD symptoms (Shalev et al., 1996). Therefore, only peritraumatic dissociation in a limited period can predict the aftermath of trauma exposure, and the identification of proper time points will benefit the early detection of PTSD susceptible individuals.

Since the prospective studies with strict control of assessing time points are hard to perform in clinical studies due to the ethical and practical limitations, the animal study is a feasible choice for the preliminary investigation. However, peritraumatic dissociation, a comprehensive syndrome which is even difficult to diagnose in humans yet (Cardena and Carlson, 2011), can hardly be mirrored in animals. Fortunately, among the symptoms of peritraumatic dissociation (acute stress disorder (ASD) in DSM-IV-tr, American Psychiatry Association, 2000), a reduction in awareness of surroundings has the potential to be assessed by acoustic startle reflex, which is an autonomic response to surrounding stimuli in most mammals. In patients with ASD, the intensity of peritraumatic dissociations was positively correlated to lower startle magnitude and more rapid habituation (Elsesser et al., 2008). Likewise, borderline personality disorder patients with high dissociative state showed a lower startle response than those with low dissociation (Ebner-Priemer et al., 2005). These results suggested that the attenuation of startle amplitude is likely to be an indicator of peritraumatic dissociation. Given that startle reflex is a cross-species response with great potential in translational studies, it can be applied in rodents to find the relationship between PTSD and peritraumatic dissociation in different time points.

In the present study, with the rat model of PTSD, the relationship between the startle response in different time points immediate after intense stress exposure and PTSD-like behaviors was examined. We hypothesized that (1) animals after intense stress would exhibit attenuated startle amplitude comparing with pre-stress level, and (2) only the startle attenuation in a specific time point would predict the development of chronic PTSD-like behaviors.

## Materials and methods

### Animals

A total of 40 adult male Sprague-Dawley rats (Vital River Laboratory Animal Technology Co. Ltd., Beijing, China) were used for the experiments. The rats (weighing 250–280 g upon arrival) were housed individually in stainless metal mesh cages (25 × 22.5 × 30 cm) with food and water *ad libitum* in a controlled temperature (20–24°C) colony room with a 12:12 h light–dark (light on at 08:00) cycle. The rats were gently handled three times before the behavioral tests. The behavioral tests started 10 days after their arrival and were conducted in the light phase (08:00–18:00). All the procedures were conducted according to the National Institutes of Health Guide for the Care and Use of Laboratory Animals, and the protocols were approved by the Research Ethics committee of Institute of Psychology, Chinese Academy of Sciences.

### General procedures

**Figure 1:**
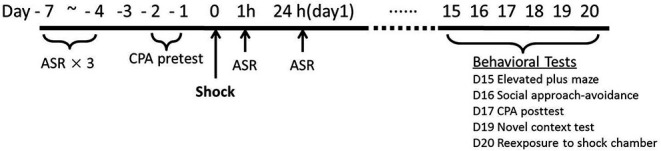
**Timeline of stress, startle assessments and PTSD-like behavioral tests**.

As depicted in Figure 1, the overall procedure included three phases: the baseline tests for startle and initial place preference, footshock stress and the measurements of startle response immediate after the stress exposure, and the assessment of PTSD-like behaviors about 2 weeks after stress.

Previous results in our laboratory found that repeated acoustic startle response (ASR) tests do not affect the amplitude of startle responses after the rats adapted the test device for enough time, so we conducted repeated ASR tests in the same animals. The animals were initially tested for ASR daily for three times. Subsequently, the rats were tested for their initial place preference to the chamber for the following conditioned place avoidance (CPA) training. Two rats were eliminated for no observable startle responses. Thirty-eight rats were assigned into Shock (*n* = 19) vs. Control (*n* = 19) group based on the baseline ASR and CPA initial preference scores.

The footshock was administered on Day 0. Peritraumatic startle responses were recorded at 1 h and 24 h after footshock stress. Chronic PTSD-like behavioral tests were conducted on Day 15 to Day 20. Given the variety and heterogeneity of PTSD symptom clusters, five PTSD-like behavioral tests were conducted in all animals about 2 weeks after stress: re-exposure to shock chamber, novel context test, social approach-avoidance, conditioned place avoidance, and elevated plus maze. Re-exposure to shock chamber was a test on the traumatic memory (Girardi et al., 2013); immobility in novel context test was a measurement to assess hyperarousal symptoms (Siegmund and Wotjak, 2007); social approach-avoidance, conditioned place avoidance, and elevated plus maze, were clinically relevant tests of avoidance and numbing symptoms (Siegmund and Wotjak, 2007; Stam, 2007).

### Footshock procedure

Footshock was given in an opaque acrylic black chamber (Beijing MacroAmbition S&T Development Co., Ltd., Beijing, China, 30 × 30 × 27 cm) with a grid floor and 200–300 lux illumination inside the chamber. The traumatic stress treatment was modified from the previous studies (Rau et al., 2005). Briefly, after a 3 min acclimation period, rats were exposed to footshocks (10 × 5 s of 1.5 mA with an intershock period of 120 ± 60 s presented randomly over 20 min) delivered by scrambled shocker (Beijing MacroAmbition S&T Development Co., Ltd., Beijing, China). The rats were kept in the chamber for another 60 s before they were returned to their home cages. Non-shock rats spent the same amount of time in the chambers but no shocks were delivered. The shock chamber was cleaned with ethanol (5%) and the bedding under the grid floor was changed for each animal.

### Behavioral tests

#### Acoustic startle response (ASR) test

The ASR test paradigm was modified from previous studies (Missig et al., 2010). The rats were acclimated to the startle system for 5 min daily for 3 days without presentation of startle stimulus. During acclimation, animals were placed in the a Plexiglas enclosure (AniLab Software & Instruments Co., Ltd., Ningbo, China, 15 × 7 × 8 cm) with constant 65 dB background white noise. The box was wiped clean between animals. The test was performed in sound attenuated startle chambers (AniLab Software & Instruments Co., Ltd., Ningbo, China). Each animal was presented with thirty 110 dB acoustic bursts (50 ms each) at random intervals (11–29 s) after 5 min habituation. ASR was detected by a piezoelectric device and recorded as 200 consecutive 1 ms recordings, starting at the onset of each startle stimulus. The startle amplitude was defined as the maximal peak-to-peak voltage that occurred during the first 200 ms after the onset of the startle-eliciting noise. Baseline startle response was measured three times daily before the stress exposure, and baseline ASR was defined as the average of the three measurements. Peritraumatic startle responses were assessed at 1 and 24 h after stress exposure.

#### Reexposure to shock chamber

As did in our previous study (Chen et al., 2012), the rats were placed back to the shock chamber without footshock for 5 min. During this period, a videotracking system (Xeye FCs, Beijing MacroAmbition S&T Development Co., Ltd., Beijing, China) was used to assess freezing time. Freezing response was defined as no movement except for those related to respiration. Freezing ratio was defined as the percent of freezing time as a percentage of total time. A larger freezing ratio indicated greater fear response.

#### Novel context test

The test arena was a black plastic box (80 × 40 × 50 cm) with 15–20 lux illumination inside. The behavioral procedures was similar to our previous study (Rogala et al., 2012), the rats were placed in the novel open field for 5 min. During this period, the Xeye FCs videotracking system was used to assess the immobility time. Immobility response was defined as no limbs movement, which indicated alertness and vigilance in the novel environments (Siegmund and Wotjak, 2007). Immobility ratio was defined as the percent of immobility time as a percentage of total time. A larger immobility ratio indicated greater hypervigilance state.

#### Social approach-avoidance (SAA) test

The social interaction arena was a black rectangle plastic box (60 × 100 × 50 cm) with 5–10 lux illumination inside. The stimulus rat (450–500 g) was put into a mesh box (30 × 15 × 30 cm) in a corner of the arena. As did in our previous study (Chen et al., 2012), the test started by introducing a test rat into an enclosed compartment (30 × 15 × 30 cm), and put this box into the opposite corner of the area where the mesh box put, leaving the sliding door of the box faced the mesh chamber of the social stimulus rat. After a 2-min habituation period, the sliding door of the enclosed box was opened, allowing the test rat to move freely in the open field for 5 min. During this period, a videotracking system (Xeye Aba, Beijing MacroAmbition S&T Development Co., Ltd., Beijing, China) was used to assess the time spent in the social zone (a 30 × 15 cm zone in front of the mesh box) and the latency to the social zone. Social time ratio was defined as the percent of social zone time as a percentage of total time. A lower social time ratio indicated greater social avoidance.

#### Conditioned place avoidance (CPA) test

The test procedure was modified based on the previous study (Li et al., 2011). Briefly, the animals were tested in a rectangular box divided into three subcompartments with different visual and tactile cues, the end subcompartments (30 × 30 × 27 cm) with black walls and the center subcompartment (10 × 30 × 27 cm) with gray wall. The floor of one end compartment was stainless grid (the same as footshock chamber floor), the floor of the other was wire mesh, and the floor of the center part was dotted with holes (2 cm). The three subcompartments were interconnected by a small opening (6 × 5 cm). The animals were placed in the center part and moved freely for 15 min. The movement of the rats was detected by infrared sensors and the time spent in different compartments was recorded by professional software (Xeye CPP, Beijing MacroAmbition S&T Development Co., Ltd., Beijing, China). CPA test were performed before and after stress exposure to measure the shift time on the shock-associated compartment. Shift score was defined as posttest time minus pretest time in the grid floor compartment. A larger shift score indicated greater shock related conditioned place avoidance.

#### Elevated plus maze (EPM) test

The Elevated plus maze (EPM) (MED associates, St. Albans, VT, USA) was composed of black Plexiglass and consisted of two open arms (50 × 10 × 0.5 cm) and two closed arms (50 × 10 × 40 cm) with all the arms extended from a center area (10 × 10 cm). The maze was elevated 50 cm above the floor and placed in a dimly lit room with a 15-W bulb. The illumination in open arms was 13–15 lux, and that in closed arm was less than 0.5 lux. As conducted in our previous study (Li et al., 2010), each animal was introduced in the center area facing a closed arm and allowed to explore freely for 5 min. The movements were recorded by a camera placed 1 m above the maze. The number of arm entries and time spent in each arm were scored by highly trained observers who were blind to experimental treatments. An arm entry was scored when all four paws of the animal entered an arm and the time duration of the rat stayed in the arm was also recorded. Open arm ratio time was calculated as the percent of time spent in the open arm as a percentage of total time spent in both the open and closed arms of the maze. A lower open arm ratio indicated greater open arm avoidance.

### Data analysis

The data on the effect of footshocks on the development of chronic PTSD-like behaviors was analyzed by independent samples student’s *t*-test or Mann–Whitney *U*-test dependent on whether the data shape of the test is Gaussian distribution or not. The data on the effect of footshock on startle response at 1 h and 24 h after stress was analyzed using paired sample student’s *t*-test.

Due to the heterogeneity in all behavioral tests, we introduced *PTSD-like score* to represent the overall development of PTSD-like behaviors (Lebow et al., 2012). Because the variance of social time ratio did not conform to a normal distribution, the raw data of five behavioral tests were transformed into rank scores (higher ranks represented higher PTSD-like behavior). PTSD-like symptoms score was the average of the five rank scores.

One-way ANOVA was conducted to analyze the alteration of ASR in the footshock group at pre-, 1 h, and 24 h after stress. Because of the obvious individual difference on the startle response, the rats in stress group were split into two subgroups based on the decline ratio of startle amplitude at 1 and 24 h after shock. The decline ratio was calculated as follows: decline ratio at 1 h = (before stress − 1 h after stress)/before stress × 100%; decline ratio at 24 h = (before stress − 24 h after stress)/before stress × 100%. Animals with decline ratio higher than 10% were categorized as decline subgroup (D), those less than 10% were in not-declined subgroup (ND). Mann–Whitney *U*-test or *t*-test were performed to examine the difference of PTSD-like symptoms score and the specific PTSD-like behaviors between the two subgroups. In addition, the animals were separated into PTSD-like subgroup (top 25%, *N* = 5) and resilient subgroup (minimum 25%, *N* = 5) according to the PTSD-like symptom score (Cohen et al., 2003), and a mixed-design two-way ANOVA was performed to examine the difference of ASR measured at different time points in the two subgroups. Finally, Spearman correlation was performed to examine the relationship between PTSD-like score and ASR decline ratio at 1 h and 24 h after stress, respectively.

## Results

### Footshocks induced post-traumatic stress disorder (PTSD)-like behaviors

**Figure 2:**
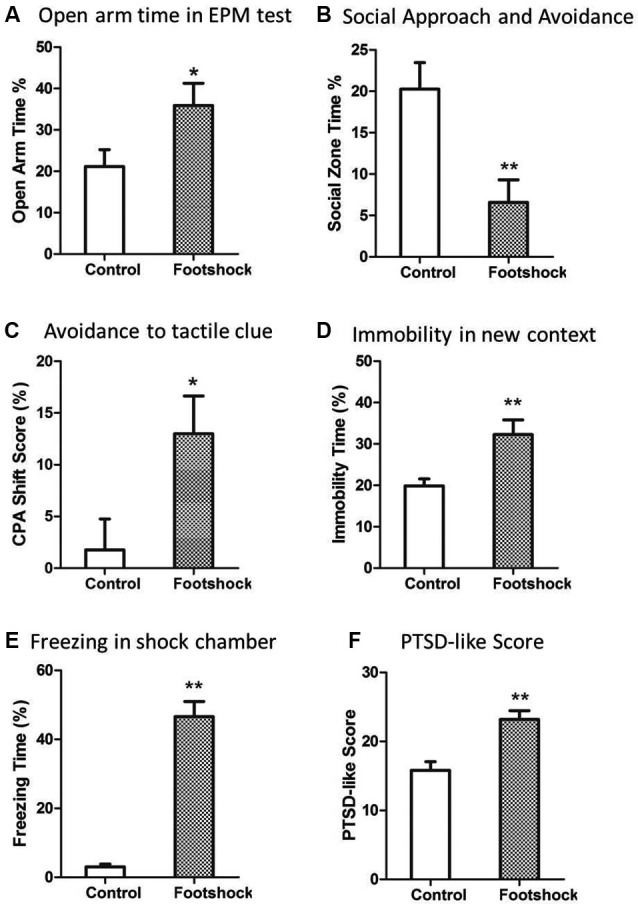
**Intense footshocks induced significant PTSD-like behaviors. (A)** Open arm time in EPM test; **(B)** Social zone time in social approach and avoidance test; **(C)** Conditioned place avoidance to shock tactile clue; **(D)** Immobility time percent in a new context; **(E)** Freezing time percent in shock chamber after re-exposure to shock context; **(F)** PTSD-like symptom score (average of five tests rank scores). * *p* < 0.05, ** *p* < 0.01.

A validated rat model of PTSD was the basis of further investigation of predictive effect of peritraumatic ASR. As expected, the rats in the footshock group developed PTSD-like behaviors in most of tests 2 weeks after stress exposure. Intense footshock stress induced significantly more avoidance of social zone (*Z* = 2.73, *p* = 0.006; Figure 2B) and shock-paired context [*t*(36) = 2.446, *p* = 0.019; Figure 2C], increased time of immobility in novel environment [*t*(36) = 2.871, *p* = 0.007; Figure 2D], and more freezing time in the shock context (*Z* = 5.27, *p* < 0.001; Figure 2E). PTSD symptom score was also much higher in the footshock group (*Z* = 3.24, *p* = 0.001; Figure 2F). Unexpectedly, animals exposed to intense footshocks displayed less avoidance towards open arm in EPM test [*t*(36) = 2.69, *p* = 0.011; Figure 2A].

### Acoustic startle response (ASR) attenuation at 1 h but not 24 h after stress predicted the development of post-traumatic stress disorder (PTSD)-like behaviors

**Figure 3:**
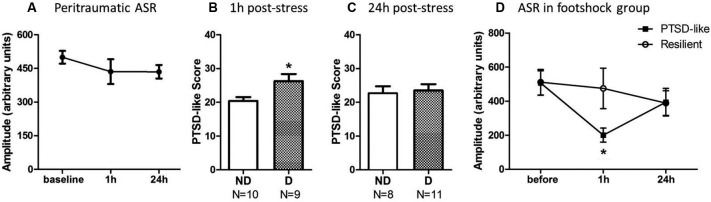
**Animals with ASR attenuation at 1 h after stress were more likely to develop severe PTSD-like behaviors 2 weeks later. (A)** The footshocked rats showed a trend on attenuation of ASR amplitude. **(B)** PTSD-like score of animals in ASR declined (D) or not-declined group (ND) according to decline ratio at 1 h after stress. **(C)** PTSD-like score of animals in ASR declined or not declined group according to decline ratio at 24 h after stress. **(D)** ASR at different time points around stress of PTSD-like and resilient individuals. * *p* < 0.05.

One-way repeated-measures ANOVA was performed to analyze the impact of stress upon startle response at 1 h and 24 h after stress. The ASR amplitude showed a trend to decrease at 1 and 24 h after the footshock (Figure 3A), but it did not reach statistically significant level [*F*(2,36) = 0.473, *p* = 0.245] since the variability of the startle response among individuals was very large.

It was necessary to compare the PTSD-like symptoms scores in the rats with or without a declined ASR immediate after stress to clarify the relationship between ASR attenuation and the development of PTSD-like behaviors. The animals were divided into ASR declined group (D) and not-declined group (ND) with the criterion that decline ratio (in method session) was more than 10%. The reason to use 10% as a cut-off point was that the declination was accounted as random fluctuation when the decline ratio was less than 10%.

The relationship of ASR attenuation at 1 h after stress and chronic PTSD-like score in footshock group was shown in Figures 3B, C. Individuals with declined ASR at 1 h after stress showed marginal significantly higher PTSD-like scores than not-declined ones [Figure 3B, not-declined (ND) *N* = 10, declined (D) *N* = 9, *Z* = 1.76, *p* = 0.076]. Table 1 summarized the difference of specific PTSD-like behavior in five behavioral tests between D and ND subgroup. No similar phenomenon was observed at 24 h after stress [Figure 3C, ND *N* = 8, D *N* = 11, *Z* = 0.548, *p* = 0.584]. It indicated that the animals with attenuated ASR at 1 h after stress are the individuals vulnerable to PTSD-like behaviors. Subsequently, we further tested whether the rats with higher PTSD-like symptom score would show a significant decrease of ASR at 1 h after stress treatment.

**Table 1:** **PTSD-like behaviors in ASR Declined and Not-Declined subjects (according to ASR in 1 h)**.

** **	**ASR Declined (*N* = 9)**	**ASR Not- Declined (*N* = 10)**
Open Arm Time (%)	30.3 ± 8.8^#^	50.6 ± 8.3
Social Zone Time (%)	2.44 ± 2.6	10.9 + 4.8
Shift Score in Shock Clue Side (%)	−12.1 ± 3.9	−13.8 ± 6.4
Immobility Time in Novel Context (%)	36.6 ± 5.3^*^	22.5 ± 2.8
Freezing Time in Shock Chamber (%)	53.2 ± 8.2	39.5 ± 4.5

Data were present as Mean ± SEM, * p = 0.021, ^#^ 0.05 < p < 0.10.

### The rats with higher post-traumatic stress disorder (PTSD)-like score showed a significant decrease of startle response at 1 h but not 24 h after stress

We separated the animals in footshock group into PTSD-like subgroup (*N* = 5, the top quarter of the high PTSD-like scores) and Resilient subgroup (*N* = 5, the minimum quarter of the PTSD-like scores) to detect whether the animals with severe PTSD-like behaviors would have the attenuated ASR at 1 h or 24 h after stress. As shown in Figure 3D, the animals in PTSD-like subgroup showed a significantly attenuated ASR at 1 h but not 24 h after the stress. It was confirmed by a two-way ANOVA analysis showing significant interaction effect (PTSD susceptibility by ASR assessment time point) [*F*(2,16) = 7.38, *p* = 0.005]. The simple effect analysis revealed a significant attenuated ASR at 1 h after stress [Figure 3D, 1 h *F*(1,8) = 5.94, *p* = 0.041], but not at 24 h after stress [*F*(1,8) = 0.00, *p* = 0.958] or before the stress [*F*(1,8) = 0.00, *p* = 0.961] between the susceptible and resilient subgroups. However, in the non-shock animals, no difference of ASR at any test time points was observed between the “susceptibility” subgroup (top quarter of the PTSD-like score) and “resilience” subgroup (the minimum quarter of the PTSD-like score), which was supported by two-way ANOVA analysis showing no interaction effect [*F*(2,16) = 0.437, *p* = 0.653] or main effect [*F*(1,8) = 2.42, *p* = 0.16]. The data suggested that the PTSD susceptibility animals showed attenuated ASR at 1 h after stress only specific to the shocked animals, consistent with the above results showing the animals with attenuated ASR at 1 h after stress predicted the development of PTSD-like behaviors.

Furthermore, spearman correlation analysis showed a positive correlation between ASR attenuation at 1 h after traumatic stress and PTSD symptoms score in the footshocked animals (*r* = 0.45, *p* = 0.05), but not in the non-shocked animals (*r* = 0.26, *p* = 0.26). The ASR decline ratio at 24 h after stress was not correlated with PTSD symptoms in either footshock group or non-shocked group (shocked *r* = −0.09, *p* = 0.71; non-shocked *r* = 0.33, *p* = 0.16). Together, the data indicated that the ASR attenuation at 1 h after stress was a key behavioral marker to predict the development of PTSD-like symptoms in shocked rats.

## Discussion

Peritraumatic dissociation, a possible predictor of PTSD, has been questioned for the inconsistent results in different time points of assessment (Bedard-Gilligan and Zoellner, 2012). The specific time window to assess the dissociation for effective prediction has not been well identified yet since the prospective clinical studies with strict control are limited. In the present study, acoustic startle response, as an objective assessment of dissociative status, was measured in different time points after stress to identify the specific time window in a rat model of PTSD. The result showed that (1) the startle response attenuated after intense footshocks, and (2) and the startle attenuation at 1 h but not 24 h after stress predicted the development of PTSD-like behaviors. To our knowledge, this is the first report finding that startle response in a specific peritraumatic time point can predict PTSD-like behaviors in a PTSD animal model with startle response as an index of peritraumatic dissociation.

The startle reflex is an involuntary defensive response to sudden and intense sensory stimulus. In rats, the startle amplitude increased after mild footshocks, while it began to negatively correlate with the shock intensity when the shock current increased to a certain level (Davis and Astrachan, 1978), and severe stress decreased the startle response (Gonzales et al., 2008). These results suggested that the ASR attenuation is a unique phenomenon after intense stress, which may protect individuals from disruptive influences of subsequent abrupt stimuli. Peritraumatic dissociation after traumatic stress was supposed to be an adaptive response that can protect the trauma victims by limiting the awareness of threatening experiences (Horowitz, 1986; Friedman, 2002; Elsesser et al., 2008). Thus, the ASR attenuation, observed in almost a half of animals at both 1 h and 24 h after stress in the present study, is assumed to indicate a special state parallel to dissociation in human.

There is also neuroendocrine evidence supporting that the alterations of startle response is related to the dissociative state. Plasma corticosterone level in rodents or cortisol release in humans was negatively correlated with ASR (Miller and Gronfier, 2006; Gonzales et al., 2008; Miller et al., 2009), and oral hydrocortisone could inhibit fear potentiated startle (Miller et al., 2011). These results indicated that the elevated cortisol or corticosterone level is one of the reason on startle attenuation immediate after severe stress. Meanwhile, the dissociative state accompanied with the elevated plasma cortisol in humans with traumatic experience (Koopman et al., 2003; Morgan et al., 2004, 2009). Moreover, an animal study with fear conditioning paradigm also found that the endogenous or exogenous high level of corticosterone can induce dissociation-like deficits in fear memory (Kaouane et al., 2012). These results supported that the attenuated ASR may be one of the behavioral manifestation of dissociative status with a common neural substrate like the elevated glucocorticoid levels after intense stress.

Moreover, the startle attenuation has been shown to share a similar neurocircuitry with the dissociative status. Neuroimaging investigations by functional MRI (fMRI) found that people in dissociative state exhibited an abnormally high activation in the medial prefrontal cortex (mPFC) and the anterior cingulate cortex (AAC; Lanius et al., 2002; Hopper et al., 2007; Felmingham et al., 2008), and an less activation in limbic system including the amygdala and anterior insula (Roder et al., 2007). ASR is under the excitatory modulation of limbic structures (Lee and Davis, 1997), mainly by bed nucleus of the stria terminalis (BNST) and the central amygdala (Walker et al., 2003), which receive inhibitory inputs from the infralimbic prefrontal cortex (Vertes, 2004). Therefore, we suppose that attenuated ASR in rat is possibly indicated a special state with an increased activation in mPFC and hyper-inhibition of limbic regions.

It is interesting that only ASR attenuation at 1 h after stress could predict the severity of chronic PTSD-like symptoms, whereas the ASR alteration at 1 day later could not (Figures 3B, C). Similarly, the startle attenuation at 1 h after stress was the main character of animals with higher PTSD-like symptom scores, comparing with the animals with lower score (Figure 3D). This phenomenon suggests that the immediate stage after stress, when the trauma memory undergoes consolidation, is crucial in the development of chronic PTSD. The startle attenuation may associate with an elevated activation in hypothalamic pituitary adrenal (HPA)-axis and mPFC, which can disrupt the fear memory consolidation and lead to an over-generalized fear memory (Kaouane et al., 2012; Xu and Sudhof, 2013). In addition, our result is consistent with the preliminary studies about early prevention of PTSD, which revealed that 1 h after stress is in a window of opportunity for intervention (Cohen et al., 2008; Zohar et al., 2011).

It is important to acknowledge that the attenuated startle in rats is only an objective measurement of the reduced awareness to surroundings, which is one of the symptoms of dissociation state. The dissociative state in humans is much complex and cannot be simply mimicked by startle attenuation in rodents. We assumed that individuals with reduced startle responses undergo an impaired fear memory formation, which contributes to the development of PTSD-like behaviors (Parsons and Ressler, 2013). Thus, the explanation about how the attenuated startle predicts the development of PTSD needs more psychophysiological and neuroendocrine evidence to support. Moreover, the present study set only two time points in assessing ASR amplitude. In further studies, the ASR tests in more time points are necessary for delineating the time course of startle alteration after acute intense stress, and providing a specific post-trauma period to assess ASR for clinical application.

Prolonged anxiety and fear-like response to the uncertain environmental stimuli even long time after the termination of traumatic stress is one of the core characteristics of PTSD symptoms. Consistent with this point, the shocked rats in the present study showed anxiety, fear or avoidance behaviors measured by social approach avoidance, re-exposure to shock chamber and conditioned place avoidance tasks. Unexpectedly, the shocked rats showed an increase on open arm time in the EPM test. It seemed that the behavioral performance of shocked rats in the EPM may not related to anxiety, since the EPM test score was not correlated with the behaviors in the SAA, shock chamber or novel environment, which were highly correlated (Data not shown). It has been established that the traumatized animals had the potential for bi-directional behavioral expression dependent on the availability of test environment (Stam, 2007), and the previous study showed that the escape behaviors increased in the shocked rats when tested in active avoidance shuttle box (Koba et al., 2001). We speculated that the shocked rats had no chance to escape when they received 10 shocks in the enclosed chamber, but in the EPM, the test environment was similar to the shuttle box where there was chance to escape from the novel and uncertain closed arm. So, the shocked rats might try to find a way to escape the enclosed area like close arm even that the open arm was potentially unsafe. It might also be related to impulsive behaviors. Further experiments with specific behavioral tasks will be needed to clarify the speculation.

Despite these limitations, the present study has important implications for the prediction of PTSD development. We set the ASR attenuation as a behavioral marker of peritraumatic dissociation to depict the relationship between chronic PTSD syndrome and the dissociation in different time points. Our results showed that the predictive effect of ASR attenuation is limited in a short period, suggesting the reduced awareness immediate after intense stress may contribute to the development of PTSD. Our findings also provide some clinical implications about the post-trauma prediction and the intervention of PTSD. It suggests that dissociation at the very immediate after trauma, which may be assessed at the arrival of emergency room, are more important than the later ones to predict PTSD, and individuals with an attenuated startle may need further intervention for the higher vulnerability of PTSD.

## Author contributions

Xinwen Dong designed the experiments, preformed the experiments, analyzed data and wrote paper; Yonghui Li designed the experiments, wrote paper and had primary responsibility for final content.

## Conflict of interest statement

The authors declare that the research was conducted in the absence of any commercial or financial relationships that could be construed as a potential conflict of interest.
